# Preoperative Diagnosis of Amyand Hernia in the Emergency Department with Point-of-care Ultrasound: A Case Report

**DOI:** 10.5811/cpcem.50746

**Published:** 2026-04-22

**Authors:** Neil Wallace, Aila Hauger

**Affiliations:** * University of Arizona, Department of Emergency Medicine, Tucson, Arizona; † University of Arizona College of Medicine - Tucson, Tucson, Arizona

**Keywords:** Amyand hernia, inguinal hernia, point-of-care ultrasound, appendicitis, case report

## Abstract

**Introduction:**

Amyand hernia is a rare condition in which the appendix is found within an inguinal hernia sac, often mimicking incarcerated or strangulated hernias. Diagnosis is typically made intraoperatively, but increasing use of point-of-care ultrasound (POCUS) is enhancing preoperative recognition.

**Case Report:**

A 46-year-old male presented with a chronic, partially reducible inguinal hernia. Point-of-care ultrasound revealed a bowel-containing hernia with fluid. Computed tomography confirmed an inflamed appendix within the sac. Laparoscopic appendectomy and open hernia repair were performed without complications.

**Conclusion:**

Early use of POCUS can expedite diagnosis of Amyand hernia, improving surgical outcomes through timely intervention.

## INTRODUCTION

Amyand hernia is a rare diagnosis in which the vermiform appendix is found within the hernia sac of an inguinal hernia. First described in the 18th century by Claudius Amyand, who performed the first successful appendectomy during hernia repair, the condition remains rare in modern surgical practice.^1^ The overall incidence of Amyand hernia ranges from 0.19–1.7% of all inguinal hernia cases, with acute appendicitis within the hernia sac occurring in just 0.07–0.13% of cases.^2^,^3^ Most Amyand hernias are diagnosed intraoperatively, as their clinical presentation typically mimics that of the more common incarcerated or strangulated inguinal hernia. This overlap often makes preoperative identification difficult, especially in the absence of systemic symptoms such as fever, leukocytosis, or peritonitis.

With the increasing availability and use of imaging modalities in the emergency department (ED), particularly point-of-care ultrasound (POCUS) and computed tomography (CT), clinicians now have improved tools to recognize rare conditions such as Amyand hernia before surgery. This case report describes a middle-aged male who presented with a chronic, partially reducible inguinal hernia and was ultimately diagnosed with Amyand hernia after POCUS and CT revealed an inflamed appendix within the hernia sac. The case highlights the growing importance of ultrasound in expediting the diagnosis and management of complex surgical conditions.

## CASE REPORT

A 46-year-old White male with a body mass index of 34 (reference range: 18.5–24.9) and a history of substance use disorder presented to the ED for possible incarcerated hernia. The patient reported several months of progressive right groin swelling and intermittent pain, with worsening symptoms over the prior three months. He noted that he first noticed the hernia five years earlier, and it had been reducible. However, over the prior 48 hours he had begun to have more scrotal pain and was unable to reduce it, causing him to present to the ED. The patient denied systemic symptoms, including fever, chills, nausea, vomiting, constipation, or abdominal pain. He had no prior abdominal surgeries and denied any urinary complaints at presentation. He had recently been released from prison and had only recently established outpatient care.

On examination, the patient was afebrile without tachycardia and non-toxic in appearance. His right scrotum was enlarged, tender, and contained a partially reducible mass. There was no overlying skin erythema or warmth. The abdomen was soft, non-tender, and non-distended. Laboratory evaluation revealed a mild leukocytosis, with white blood cells counted to 12,000 per microliter (μL) (reference range: 4,500–11,000/μL) with neutrophilic predominance. The remainder of the complete blood count was within normal limits. Electrolytes, renal function, liver enzymes, and urinalysis were unremarkable. A POCUS of the groin demonstrated a bowel-containing right inguinal hernia measuring 5 cm × 5 cm × 5 cm, with surrounding fluid extending into the scrotum, as well as a non-compressible, hyperemic, blind-ended tubular structure (Image).

The hernia was noted to be partially reducible during the scan. Given the ultrasound findings, CT of the abdomen and pelvis with contrast was obtained. The CT revealed a right inguinal hernia containing the distal ileum, cecum, and appendix. The appendix measured 9 mm in diameter and was surrounded by fat stranding and fluid within the hernia sac, concerning for acute appendicitis. There was no evidence of bowel obstruction or free air.

The patient was evaluated by the surgical team and admitted with a plan for operative intervention. He was made nil per os, started on intravenous fluids, and received preoperative antibiotics. In the operating room, laparoscopy revealed an acutely inflamed appendix within the hernia sac, along with the cecum and ileum. The hernia demonstrated both direct and indirect components, and the floor of the inguinal canal was obliterated. A laparoscopic appendectomy was performed, followed by open right inguinal hernia repair with mesh placement. There were no intraoperative complications, and estimated blood loss was 100 mL.

*CPC-EM Capsule*

What do we already know about this clinical entity?

*Amyand’s hernia is an inguinal hernia containing the appendix, occurring in ~1% of inguinal hernias. It’s rarely diagnosed preoperatively, and may either be normal or inflamed*.

What makes this presentation of disease reportable?

*This case report shows an Amyand’s hernia diagnosed by emergency department performed point-of-care ultrasound, highlighting important sonographic features*.

What is the major learning point?

*Inguinal hernias may contain a variety of abdominal structures, including the appendix*.

How might this improve emergency medicine practice?

*Identifying an inguinal hernia at risk of complication from bedside reduction is important, and point-of-care ultrasound may help identify high risk features*.

Gross pathology confirmed acute appendicitis, with a 6.2-cm vermiform appendix showing congested serosa and fibrinous exudate. No perforation or fecaliths were identified. The patient was transferred to the surgical floor postoperatively and had an uncomplicated hospital course. He was discharged the following day with activity restrictions, instructions for follow-up, and return precautions.

At his two-week outpatient follow-up, the patient reported no fever, pain, or drainage from the surgical sites. On exam, his incisions were clean, dry, and intact with no signs of infection. He was tolerating his diet, having regular bowel movements, and voiding without difficulty.

## DISCUSSION

Amyand hernia represents a rare surgical finding, characterized by the presence of the vermiform appendix within an inguinal hernia sac. The incidence of Amyand hernia is reported to range from 0.19–1.7% of inguinal hernia cases and, when appendicitis is involved, it becomes even less common with estimates ranging from 0.07%–0.13%.^2^,^3^ A retrospective review by D’Alia et al identified Amyand hernia in 0.6% of 1,341 inguinal hernias, all occurring on the right side and exclusively in male patients.^4^ As previously mentioned, Amyand hernia holds a unique place in surgical history. In 1735, Claudius Amyand performed what is recognized as the second documented appendectomy on an 11-year-old patient whose inflamed appendix was located within an inguinal hernia sac, later giving rise to the eponymous condition.^1^ This operation marked both the initial description and successful surgical management of Amyand hernia, as well as one of the earliest documented appendectomies in medical literature.

In this case, the chronicity of symptoms without overt signs of systemic infection made the clinical picture more ambiguous, underscoring the value of early imaging, particularly POCUS, in the ED. Ultrasound is increasingly used in the ED as a first-line modality for evaluating scrotal and inguinal pathology due to its accessibility, lack of radiation, and ability to provide dynamic assessment. The American College of Radiology recommends ultrasound as the preferred initial imaging modality for suspected groin hernia, citing sensitivities as high as 97% when performed by skilled operators.^5^ While there are varying levels of competency with POCUS, there have been several studies that show emergency physicians can perform POCUS to reliably confirm the presence of a hernia sac, identify features of incarceration or strangulation (such as non-reducible bowel, free fluid, or absent Doppler flow), and detect associated small bowel obstruction, thus expediting surgical decision-making and risk stratification in the emergency setting.^6^,^7^ However, it is understandable that identifying Amyand hernia on POCUS is unlikely expected of novice sonographers. Additional systematic reviews and meta-analyses support the reliability of ultrasound in diagnosing both overt and occult groin hernias, as well as in accurately distinguishing between inguinal and femoral types.^8^

Of note: The chart abstractors were not blinded to the patient outcomes and this report. In our patient, POCUS was instrumental in identifying a bowel-containing inguinal hernia with associated fluid and signs of inflammation, prompting further investigation with CT and early surgical consultation. The majority of Amyand hernia cases are identified intraoperatively, and this pattern has been well documented in the medical literature. Multiple systematic reviews and case series consistently report that the clinical presentation of Amyand hernia is indistinguishable from that of an incarcerated or strangulated inguinal hernia, making preoperative diagnosis challenging. One systematic review found that only 23.1% of cases were diagnosed preoperatively, most often with the aid of imaging such as ultrasound or CT, while the remainder were discovered during surgery for presumed incarcerated hernia.^9^ Similarly, other series and reviews emphasize that intraoperative identification is the norm, with preoperative suspicion being low due to non-specific clinical findings.^2^,^9^,^10^

While our patient ultimately underwent CT for further characterization of the rare finding of Amyand hernia, POCUS assisted with expediting surgical referral and confirming the diagnosis of an incarcerated hernia early in the clinical course. Ultimately, the surgical team decided to pursue operative management given the free fluid in the hernia sac and risk of perforation with bedside reduction. In patients with an inconclusive or atypical physical exam, POCUS can facilitate timely surgical consultation when positive and may help avoid unnecessary radiation exposure from CT imaging when negative. In this case, the diagnostic utility of POCUS was highlighted by the early surgical consultation, which ultimately allowed for early surgical intervention. This is important as delays in emergent hernia repair have been associated with increased complication rates, including prolonged operative time, extended postoperative hospital stay, and higher rates of reoperation.^11^

The hernia sac itself is formed by peritoneum protruding through an abdominal wall defect, and the herniated contents are typically mobile and reducible unless incarcerated or strangulated.^12^ Typical hernia contents include small bowel, which is identified by its characteristic peristalsis and fluid- or gas-filled loops. In contrast, preperitoneal fat appears as a homogeneous, moderately hyperechoic, non-vascular, non-peristaltic structure that does not share the layered appearance of bowel and typically shows minimal or no response to Valsalva maneuvers. Omental fat is also hyperechoic but generally more heterogeneous and lobulated than preperitoneal fat. It may contain small vessels visible on color Doppler and lacks the tubular morphology and peristaltic motion of bowel.^12^ Less commonly, the urinary bladder may herniate, presenting as a fluid-filled, anechoic, and non-peristaltic structure that may change with voiding.^13^ In female patients, the ovary or fallopian tube can herniate and may be seen as complex adnexal structures with internal follicles and varying Doppler flow; the ovary may appear hypoechoic or heterogeneous, depending on vascular compromise.^14^ Rarely, a Meckel diverticulum (Littre hernia) may be present within the sac.

Point-of-care ultrasound plays additional roles in the emergency evaluation of inguinal hernias, particularly in differentiating hernia contents and identifying high-risk features. The decision to reduce a hernia is often a clinical one, incorporating history and physical exam. Point-of-care ultrasound is an adjunct that helps identify high-risk hernias that should not be reduced. These include hernias with features of strangulation or ischemic bowel. While success of non-operative reduction of Amyand hernia is unknown due to its rare presentation, care should be taken with certain high-risk features that might predispose to rupture, such as a hyperemic appendix with surrounding free fluid.^10^ Signs concerning for strangulation include non-compressible bowel, bowel wall thickening, free fluid in the hernia sac, and absent or reduced Doppler flow. These findings should prompt immediate surgical consultation rather than manual reduction, as reduction of non-viable bowel may result in perforation, peritonitis, or sepsis.^15^

## CONCLUSION

Our case illustrates how the early integration of POCUS into the evaluation of groin complaints can both narrow the differential and expedite appropriate surgical care. Emergency physicians should maintain a high index of suspicion and incorporate ultrasound early in the evaluation of groin complaints, particularly in cases with chronicity, atypical features, or inconclusive physical exam findings. Incorporating early ultrasound can facilitate earlier recognition, better communication with surgical teams, and improved patient outcomes.

## Figures and Tables

**Image:**
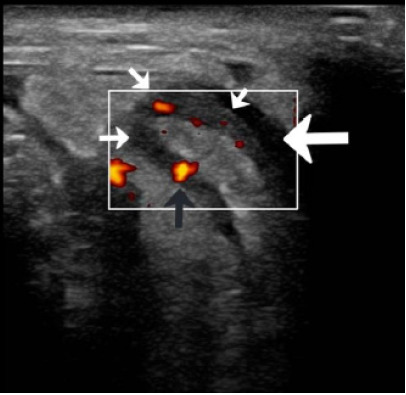
A blind-ended tubular structure, the edematous appendiceal wall (thin white arrows) with enhanced Doppler flow (black arrow) and free fluid (thick white arrow).
